# Draft Genome Sequence of *Brevibacillus* sp. Strain BAB-2500, a Strain That Might Play an Important Role in Agriculture

**DOI:** 10.1128/genomeA.00021-13

**Published:** 2013-02-28

**Authors:** M. N. Joshi, A. Sharma, A. S. Pandit, R. V. Pandya, A. K. Saxena, S. B. Bagatharia

**Affiliations:** Gujarat State Biotechnology Mission, Department of Science & Technology, Udyog Bhavan, Gandhinagar, Gujarat, India

## Abstract

A Gram-positive bacterium, *Brevibacillus* sp. strain BAB-2500, was isolated as a lab contaminant in Gandhinagar, Gujarat, India. The draft genome (5.3 Mb) of the strain possesses genes for the reduction of arsenate and aluminum. These findings might provide insights into the utilization of this strain for improving crop production.

## GENOME ANNOUNCEMENT

 The genus *Brevibacillus* was established within the family *Paenibacillaceae* by Shida et al. (1). He reclassified nine species of the genus *Bacillus*, i.e., *Bacillus brevis* (2), *Bacillus laterosporus* (3) *Bacillus thermoruber* (4), *Bacillus agri* (5), *Bacillus centrosporus* (5), *Bacillus choshinensis* (6), *Bacillus borstelensis* (7), *Bacillus formosus* (7), and *Bacillus reuszeri* (7), and placed them within a new genus, *Brevibacillus* (1). Subsequently, 8 validly named species were placed taxonomically within this genus: *Brevibacillus invocatus* (8), *Brevibacillus limnophilus* (9), *Brevibacillus levickii* (10), *Brevibacillus ginsengisoli* (11), *Brevibacillus panacihumi* (12), *Brevibacillus fluminis* (13), *Brevibacillus aydinogluensis* (14), and *Brevibacillus nitrificans* (15). Members of this genus are reported to be rod-shaped, Gram-positive or Gram-variable, motile by means of peritrichous flagella, and strictly aerobic, with G+C content ranging from 42.8 to 57.4%. Earlier studies have shed light on the agricultural importance of a few members of this genus, for use as biocontrol agents (16), denitrifying bacterium (11), etc. Thus, *Brevibacillus* sp. strain BAB-2500, isolated as a lab contaminant, was subjected to whole genome sequencing for genomic study; this can add more knowledge about the agricultural as well as other potential applications of the members of this genus.

Genome sequencing of the strain was done with a high-throughput Ion Torrent Personal Genome machine with Ion Torrent Server (Torrent suite v3.2), and total data of 1,581,318 paired-end reads with 22.46× coverage (mean length read is 90 bp with highest read of 200 bp) were obtained. *De novo* assembly was performed using the MIRA-3 assembler (v3.1.0). The annotation of the genome was performed using the NCBI Prokaryotic Genomes Automatic Annotation Pipeline (PGAAP) (http://www.ncbi.nlm.nih.gov/genomes/static/Pipeline.html) utilizing GeneMark, Glimmer, and tRNAscan-SE tools (17), and using the Rapid Annotations using Subsystems Technology (RAST) server with the SEED database (18).

The total length of the genome was found to be 5,38,6487 bp, allocated into 329 contigs having >500 bp each (scaffold N_50_; largest contigs, 48,866 bp) and 52 contigs ≤500 bp. The G+C content was 53.5%. The draft genome of the strain BAB-2500 harbored 5,457 genes that encode 5,349 protein-coding genes, 94 transfer RNAs, and 14 ribosomal RNAs.

The genome harbored genes for the biosynthesis of vitamins B_12_ and B_5_. Transporters for multidrug resistance, potassium uptake, and arsenical pump efflux were identified within the genome. Genes for the biosynthesis of various sugars and the degradation of various aminosugars were also annotated. The loci of the CRISPR-associated endoribonuclease (CaS2) gene were present in the genome, which might play a role in providing the acquired immunity to species. The strain also exhibited an arsenate reductase enzyme that catalyzes the reduction of the toxic compound arsenate to arsenite. Cystathionine beta-lyase protein, which significantly contributes to aluminum resistance, was also predicted within the genome. These findings suggest that the strain might play a vital role in the improvement of crop production in metal-contaminated soil, as well as in acidic soil. Therefore, the draft genome of the species provides insights into the functional characterization of significant genes within the genome.

### Nucleotide sequence accession number.

The draft genome sequence of *Brevibacillus* sp. BAB-2500 has been deposited at GenBank under the accession no. AOBR00000000.

## References

[1] Shida O Takagi H Kadowaki K Komagata K 1996 Proposal for two new genera, *Brevibacillus* gen. nov. and *Aneurinibacillus* gen. nov. Int. J. Syst. Bacteriol. 46:939–946 8863420 10.1099/00207713-46-4-939

[2] Migula W 1900 System der bakterien, vol 2 Gustav Fischer, Jena, Germany

[3] Claus D Berkeley RCW 1986 Genus *Bacillus* Cohn 1872, p 1105–1140 Murray RGE Sneath PHA Sharpe ME Holt JG, Bergey’s manual of systematic bacteriology, vol 2. The Williams & Wilkins Co., Baltimore, MD.

[4] Manachini PL Fortina MG Parini C Craveri R 1985 *Bacillus thermoruber* sp. nov., nom. rev., a red-pigmented thermophilic bacterium. Int. J. Syst. Bacteriol. 35:493–496

[5] Nakamura LK 1993 DNA relatedness of *Bacillus brevis* Migula 1900 strains and proposal of *Bacillus agri* sp. nov., nom. rev., and *Bacillus centrosporus* sp. nov., nom. rev. Int. J. Syst. Bacteriol. 43:20–25

[6] Takagi H Shida O Kadowaki K Komagata K Udaka S 1993 Characterization of *Bacillus brevis*, with descriptions of *Bacillus migulanus* sp. nov., *Bacillus choshinensis* sp. nov., *Bacillus parabrevis* sp. nov, and *Bacillus galuctophilus* sp. Int. J. Syst. Bacteriol. 43:221–231 8494737 10.1099/00207713-43-2-221

[7] Shida O Takagi H Kadowaki K Nakamura LK Komagata K 1995 Proposal of *Bacillus reusten* sp. nov. *Bacillus formosus* sp. nov., nom. rev., and *Bacillus horstelensis* sp. nov., nom. rev. Int. J. Syst. Bacteriol. 45:93–100

[8] Logan NA Forsyth G Lebbe L Goris J Heyndrickx M Balcaen A Verhelst A Falsen E Ljungh A Hansson HB De vos P 2002 Polyphasic identification of *Bacillus* and *Brevibacillus* strains from clinical, dairy and industrial specimens and proposal of *Brevibacillus invocatus* sp. Int. J. Syst. Evol. Microbiol. 52:953–966 12054263 10.1099/00207713-52-3-953

[9] Goto K Fujita R Kato Y Asahara M Yokota A 2004 Reclassification of *Brevibacillus brevis* strains NCIMB 13288 and DSM 6472 (= NRRL NRS-887) as *Aneurinibacillus danicus* sp. nov. and *Brevibacillus limnophilus* sp. nov. Int. J. Syst. Evol. Microbiol. 54:419–427 15023954 10.1099/ijs.0.02906-0

[10] Allan RN Lebbe L Heyrman J De vos P Buchanan CJ Logan A 2005 *Brevibacillus levickii* sp. nov. and *Aneurinibacillus terranovensis* sp. nov., two novel thermoacidophiles isolated from geothermal soils of northern Victoria Land, Antarctica. Int. J. Syst. Evol. Microbiol. 55:1039–1050 15879231 10.1099/ijs.0.63397-0

[11] Baek SH Im WT Oh HW Lee JS Oh HM Lee ST 2006 *Brevibacillus ginsengisoli* sp. nov., a denitrifying bacterium isolated from soil of a ginseng field. Int. J. Syst. Evol. Microbiol. 56:2665–2669 17082408 10.1099/ijs.0.64382-0

[12] Kim MK Sathiyaraj S Pulla RK Yang DC 2009 *Brevibacillus panacihumi* p. nov., a β-glucosidase-producing bacterium. Int. J. Syst. Evol. Microbiol. 59:1227–1231 19406823 10.1099/ijs.0.001248-0

[13] Choi MJ Bae JY Kim KY Kang H Cha CJ 2010 *Brevibacillus fluminis* sp. nov., isolated from sediment of estuarine wetland. Int. J. Syst. Evol. Microbiol. 60:1595–1599 19700454 10.1099/ijs.0.012351-0

[14] Inan K Canakci S Belduz AO Sahin F 2012 *Brevibacillus aydinogluensis* sp. nov., a moderately thermophilic bacterium isolated from Karakoc hot spring. Int. J. Syst. Evol. Microbiol. 62:849–855 21622837 10.1099/ijs.0.031914-0

[15] Takebe F Hirota K Nodasaka Y Yumoto I 2012 *Brevibacillus nitrificans* sp. nov., a nitrifying bacterium isolated from a microbiological agent for enhancing microbial digestion in sewage treatment tanks. Int. J. Syst. Evol. Microbiol. 62:2121–2126 22039003 10.1099/ijs.0.032342-0

[16] de Oliveira EJ Rabinovitch L Monnerat RG Jannotti Passos LK Zahner V 2004 Molecular characterization of *Brevibacillus laterosporus* and its potential use in biological control. Appl. Environ. Microbiol. 70:6657–6664 15528531 10.1128/AEM.70.11.6657-6664.2004 PMC525142

[17] Aziz RK Bartels D Best AA DeJongh M Disz T Edwards RA Formsma K Gerdes S Glass EM Kubal M Olsen GJ Olson R Osterman AL Overbeek RA McNeil LK Paarmann D Paczian T Parrello B Pusch GD Reich C Stevens R Vassieva O Vonstein V Wilke A Zagnitko O 2008 The RAST server: rapid annotations using subsystems technology. BMC Genomics 9:75 18261238 10.1186/1471-2164-9-75 PMC2265698

[18] Disz T Akhter S Cuevas D Olson R Overbeek R Vonstein V Stevens R Edwards RA 2010 Accessing the SEED genome databases via Web services API: tools for programmers. BMC Bioinformatics 11:319 20546611 10.1186/1471-2105-11-319 PMC2900279

